# Real-world outcomes of first-line treatment with HLX02 versus reference trastuzumab plus pertuzumab in HER2-positive metastatic breast cancer

**DOI:** 10.1093/oncolo/oyaf183

**Published:** 2025-07-16

**Authors:** Ying Yan, Ruyan Zhang, Yanling Li, Lijun Di, Xinyu Gui, Hanfang Jiang, Xu Liang, Bin Shao, Guohong Song, Huiping Li

**Affiliations:** Key Laboratory of Carcinogenesis and Translational Research (Ministry of Education/Beijing), Department of Breast Oncology, Peking University Cancer Hospital and Institute, Beijing 100142, China; Key Laboratory of Carcinogenesis and Translational Research (Ministry of Education/Beijing), Department of Breast Oncology, Peking University Cancer Hospital and Institute, Beijing 100142, China; Key Laboratory of Carcinogenesis and Translational Research (Ministry of Education/Beijing), Department of Breast Oncology, Peking University Cancer Hospital and Institute, Beijing 100142, China; Key Laboratory of Carcinogenesis and Translational Research (Ministry of Education/Beijing), Department of Breast Oncology, Peking University Cancer Hospital and Institute, Beijing 100142, China; Key Laboratory of Carcinogenesis and Translational Research (Ministry of Education/Beijing), Department of Breast Oncology, Peking University Cancer Hospital and Institute, Beijing 100142, China; Key Laboratory of Carcinogenesis and Translational Research (Ministry of Education/Beijing), Department of Breast Oncology, Peking University Cancer Hospital and Institute, Beijing 100142, China; Key Laboratory of Carcinogenesis and Translational Research (Ministry of Education/Beijing), Department of Breast Oncology, Peking University Cancer Hospital and Institute, Beijing 100142, China; Key Laboratory of Carcinogenesis and Translational Research (Ministry of Education/Beijing), Department of Breast Oncology, Peking University Cancer Hospital and Institute, Beijing 100142, China; Key Laboratory of Carcinogenesis and Translational Research (Ministry of Education/Beijing), Department of Breast Oncology, Peking University Cancer Hospital and Institute, Beijing 100142, China; Key Laboratory of Carcinogenesis and Translational Research (Ministry of Education/Beijing), Department of Breast Oncology, Peking University Cancer Hospital and Institute, Beijing 100142, China

**Keywords:** metastatic breast cancer, HER2 positive, trastuzumab, pertuzumab, biosimilar

## Abstract

**Background:**

HLX02 (Zercepac®) is the first trastuzumab biosimilar manufactured in China. This study presents the first real-world comparison of HLX02 with reference trastuzumab (RTZ) plus pertuzumab and chemotherapy as the first-line treatment for HER2-positive metastatic breast cancer (MBC) patients.

**Methods:**

Medical data of patients with HER2-positive MBC who received HLX02 or RTZ, both combined with pertuzumab and various chemotherapies as the first-line therapy at Beijing Cancer Hospital from January 2019 to August 2023 were reviewed retrospectively. The survival outcomes, efficacy, and adverse events were analyzed.

**Results:**

In total, 118 patients were included in this study retrospectively, among whom 66 patients received RTZ and 52 received HLX02. No significant difference was observed in progression-free survival (PFS) between the groups (median PFS: 22.0 months for RTZ vs. 19.0 months for HLX02, *P* = .832). Additionally, the objective response rate, disease control rate, and safety profiles were similar across both groups. Of all 118 patients, 20 (16.9%) patients experienced progression in the central nervous system (CNS), with a median time to CNS progression of 15.0 months (95% confidence interval, CI, 12.8–17.2).

**Conclusion:**

The real-world data suggested that both HLX02 and RTZ, when combined with pertuzumab and various chemotherapy regimens, offer comparable efficacy and safety as first-line treatments for HER2-positive advanced breast cancer patients in China.

**Implications for Practice**

This real-world data suggested that both HLX02 and reference trastuzumab, when combined with pertuzumab and various chemotherapy regimens, offer comparable efficacy and safety as the first-line treatment for human epidermal growth factor receptor 2 (HER2)-positive metastatic breast cancer patients. Overall, the progression-free survival outcomes were consistent with the CLinical Evaluation Of Pertuzumab And TRAstuzumab trial for both groups, and no new safety signals were identified. This suggested that HLX02 may provide another option of HER2-targeted therapy combined with pertuzumab for Chinese patients. The benefit regarding long-term survival need to be further verified.

## Introduction

Approximately 15% to 20% of breast cancer patients exhibit overexpression of human epidermal growth factor receptor 2 (HER2) protein and/or amplification of its gene.^1^ The randomized phase III CLinical Evaluation Of Pertuzumab And TRAstuzumab (CLEOPATRA) trial evaluated the efficacy of adding pertuzumab to trastuzumab and docetaxel as a new first-line standard.^2^ It reported significant improvements in both progression-free survival progression-free survival (PFS) and overall survival (OS) with dual HER2 blockade. Similar outcomes were observed in the Chinese bridging PUFFIN trial.^3^ Nevertheless, the high cost of trastuzumab (Herceptin, F. Hoffmann–La Roche/Genentech) and pertuzumab (Perjeta, F. Hoffmann–La Roche/Genentech) limit their accessibility and use among eligible patients, especially in developing countries such as China. Biosimilars, offering competitively lower-cost alternatives to original biologics, might improve accessibility in cancer care. HLX02 (Zercepac®, Henlius, Inc.), the first globally evaluated trastuzumab biosimilar manufactured in China, has demonstrated similar safety, efficacy, tolerability, and immunogenicity to reference trastuzumab (RTZ, Herceptin).^4^ Based on the results of the phase III trial comparing HLX02 with RTZ combined with docetaxel in patients with HER2-positive metastatic breast cancer (MBC), HLX02 has been approved in China as a trastuzumab biosimilar. However, real-world studies investigating the efficacy and safety of HLX02 in combination with pertuzumab are limited.

In this real-world study, we evaluated the efficacy and safety of trastuzumab biosimilar HLX02 and RTZ (Herceptin) in combination with pertuzumab as the first-line therapy for HER2-positive MBC. This analysis provides evidence-based guidance for trastuzumab biosimilar in real-world clinical practice.

## Materials and methods

Clinical data was collected retrospectively from the medical records of patients with HER2-positive MBC who received first-line treatment with trastuzumab and pertuzumab combined with chemotherapies between January 2019 and August 2023 at Beijing Cancer Hospital. The choice of trastuzumab included HLX02 or Herceptin, which was determined by physicians and patients.

The primary outcome was real-world PFS. Secondary outcomes were objective response rate (ORR), disease control rate (DCR), and safety. Real-world tumor response assessment was performed by 2 authors and a study radiologist in our hospital with a Good Clinical Practice (GCP) certificate based on imaging, clinical, or laboratory evidence according to the Response Evaluation Criteria in Solid Tumors. ORR/DCR was calculated, real-world PFS and time to the central nervous system (CNS) progression from first-line therapy were estimated using the Kaplan–Meier curve. Adverse events (AEs) during the treatment were graded according to the National Cancer Institute Common Terminology Criteria for Adverse Events (NCI CTCAE), version 5.0. All data were analyzed using the Statistical Package for Social Sciences software version 26.0.

## Results

In total, 118 patients were included in this study, among whom 66 patients were treated with RTZ and 52 patients with HLX02. The baseline characteristics are summarized in Table 1. Approximately one-third of the patients were aged ≥ 60 years. The proportion of patients with de novo stage IV disease was comparable between the 2 groups (54.5% for RTZ group vs. 61.5% for HLX02 group). The majority of patients in both treatment groups had visceral metastases at diagnosis, with a slightly lower incidence in the HLX02 group (57.7%) compared to the RTZ group (71.2%). A small percentage of both groups had previously received trastuzumab in the (neo)adjuvant setting (13.6% for RTZ group vs. 13.5% for HLX02 group).

**Table 1.** Baseline characteristics of patients with HER2-positive advanced breast cancer.

Characteristics	Total (*n* = 118)	Herceptin (*n* = 66)	HLX02 (*n *= 52)
Age (years)			
Median (range)	56 (31–73)	56 (31–73)	56 (31–70)
< 60, *n* (%)	80 (67.8)	45 (68.2)	35 (67.3)
≥ 60, *n* (%)	38 (32.2)	21 (31.8)	17 (32.7)
Hormone receptor status, *n* (%)			
ER and/or PR positive	53 (44.9)	34 (51.5)	19 (36.5)
ER and PR negative	65 (55.1)	32 (48.5)	33 (63.5)
Disease status, *n* (%)			
De novo	68 (57.6)	36 (54.5)	32 (61.5)
Recurrent	50 (42.4)	30 (45.5)	20 (38.5)
Visceral metastases, *n* (%)	77 (65.3)	47 (71.2)	30 (57.7)
Liver	51 (43.2)	33 (50.0)	18 (34.6)
Lung	40 (33.9)	25 (37.9)	16 (30.8)
Brain	5 (4.2)	2 (3.0)	3 (5.8)
Prior neo-/adjuvant therapy with trastuzumab			
Yes	16 (13.6)	9 (13.6)	7 (13.5)
No	102 (86.4)	57 (86.4)	45 (86.5)
Chemotherapy regimens with trastuzumab + pertuzumab			
Taxanes	111 (94.1)	62 (94.0)	49 (94.2)
Nab-paclitaxel	54 (45.8)	30 (45.5)	24 (46.2)
Docetaxel	37 (31.4)	28 (42.4)	9 (17.3)
Paclitaxel	20 (16.9)	4 (6.0)	16 (30.7)
Vinorelbine	5 (4.2)	2 (3.0)	3 (5.8)
Capecitabine	2 (1.7)	2 (3.0)	0 (0)

Abbreviations: ER, estrogen receptor; HER2, human epidermal growth factor receptor 2; PR, progesterone receptor.

Taxanes were the most commonly used chemotherapy regimen in both groups, with 94.0% of the RTZ group and 94.2% of the HLX02 group, others included vinorelbine (4.2%) and capecitabine(1.7%).

## Clinical outcomes

**Figure 1.**
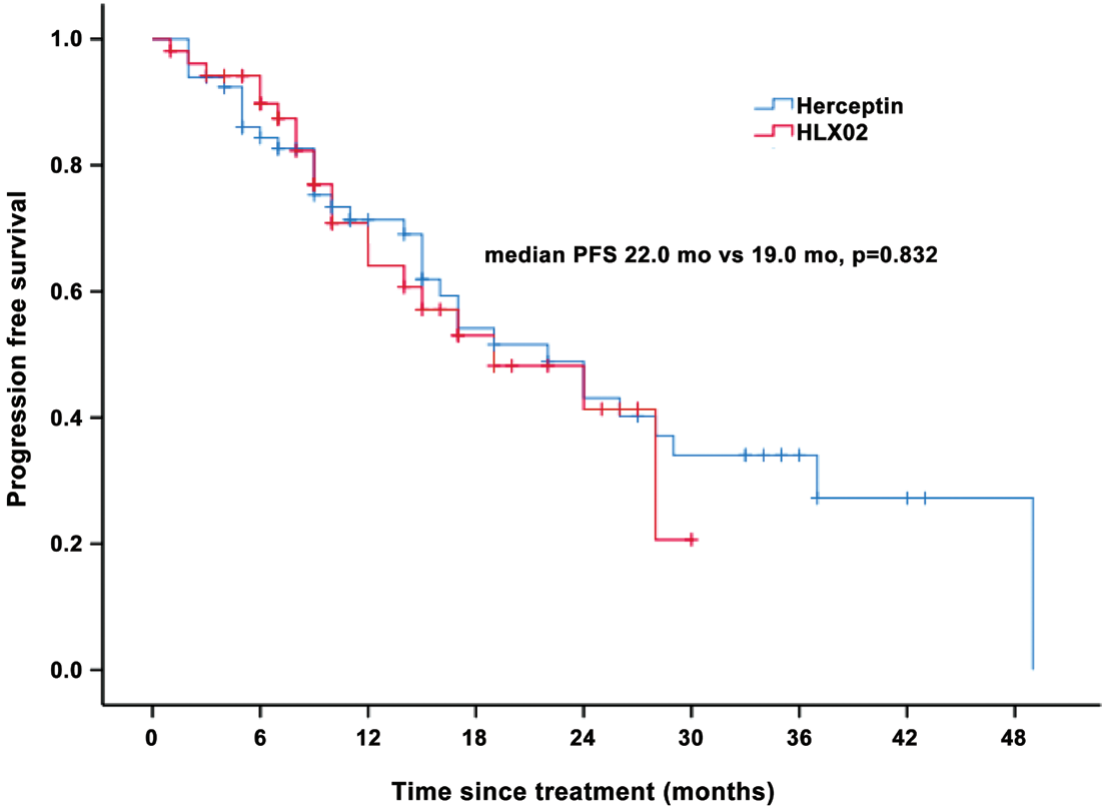
Progresssion-free survival (PFS) between Herceptin and HLX02.

During a median follow-up of 15.0 months (range, 1.0-58.0 months; 95% CI, 12.6-17.4), the median PFS for all patients of this study was 22.0 months (95% CI, 16.3-27.7). The median PFS was comparable between the RTZ group (22.0 months) and the HLX02 group (19.0 months; *P* = .832) (Figure 1). In subgroup analysis, survival outcomes were similar across different clinicopathological characters, including hormonal receptor status, disease status (de novo or recurrent), and metastatic site (visceral or non-visceral).

In the analysis of 118 patients with measurable disease, the ORR showed no difference between the 2 groups, with 72.7% in the RTZ group and 75.0% in the HLX02 group achieving a confirmed objective response. The DCR, defined as the percentage of patients who achieved a complete response (CR), partial response (PR), or stable disease (SD), was 93.9% in the RTZ group and 94.2% in the HLX02 group.

## CNS progression

Among 118 patients, 20 (16.9%) experienced CNS progression (15 in RTZ and 5 in HLX02 group), including 18 with newly diagnosed CNS metastasis and 2 with progression of preexisting CNS metastasis at baseline. Most of them (16 patients, 13.6%) were isolated to the CNS without extracranial progression. The median time from the initiation of first-line therapy to CNS progression was 15.0 months (95% CI, 12.8-17.2).

After CNS progression under trastuzumab and pertuzumab, the majority (16/20 patients, 80%) received systemic treatments in combination with CNS-directed local therapy, while a minority (4/20 patients, 20%) received systemic treatments alone. Among the 20 patients with CNS progression, 16 patients switched to a pyrotinib-based systemic treatment, 3 patients continued trastuzumab and pertuzumab combined with local therapy, and 1 patient switched to Trastuzumab deruxtecan (T-DM1).

## Safety

Among the 118 patients, 75 (63.6%) reported AEs, and 9 (7.6%) experienced grade ≥ 3 AEs (shown in Table 2). The most common AEs of any grade were leucopenia (31.4%), increased alanine aminotransferase (ALT, 31.4%), and increased aspartate aminotransferase (AST, 22.9%). Although these AEs were commonly associated with chemotherapy regimens, there were some differences in their incidence between the 2 groups. A higher incidence of grade ≥ 3 neutropenia occurred in the RTZ group (10.6% vs. 0), but no febrile neutropenia was reported. Additionally, no significant decreases in left ventricular ejection fraction were reported in either group.

**Table 2.** Treatment related adverse events.

Event, *n* (%)	Total (*n* = 118)		Herceptin (*n* = 66)		HLX02 (*n* = 52)	
	Any grade	Grade 3–4	Any grade	Grade 3–4	Any grade	Grade 3–4
Any event	75 (63.6)	9 (7.6)	52 (78.7)	8 (12.1)	23 (44.2)	1 (1.9)
Leucopenia	37 (31.4)	6 (5.1)	28 (42.4)	6 (9.1)	9 (17.3)	0
Neutropenia	25 (21.2)	7 (5.9)	22 (33.3)	7 (10.6)	3 (5.8)	0
Anemia	19 (16.1)	0	15 (22.7)	0	4 (7.7)	0
Platelet count decreased	2 (1.7)	0	2 (3.0)	0	0	0
Increased ALT	37 (31.4)	0	21 (31.8)	0	16 (30.8)	0
Increased AST	27 (22.9)	1 (0.8)	19 (28.8)	0	8 (15.4)	1 (1.9)
Blood bilirubin increased	25 (21.2)	1 (0.8)	17 (25.8)	1 (1.5)	8 (15.4)	0

Abbreviations: ALT, alanine aminotransferase; AST, aspartate aminotransferase.

## Discussion

We retrospectively analyzed real-world, single-center clinical data from patients treated with the regimen based on trastuzumab and pertuzumab as the first-line therapy for HER2-positive MBC. In addition, we assessed the equivalence in efficacy and safety between RTZ (Herceptin) and HLX02, both in combination with pertuzumab. The survival outcomes observed in this study were consistent with those reported in previous studies of trastuzumab plus pertuzumab in the metastatic setting, including several real-world studies.

It revealed that the median PFS (22.0 months for Herceptin vs. 19.0 months for HLX02; *P* = .832) and ORR (72.7% for Herceptin vs. 75.0% for HLX02) were comparable between the 2 groups. This real-world study provides an evidence-based foundation for the clinical replacement of biosimilars in China.

CNS is one of the most common metastatic sites of HER2-positive MBC, which is associated with a poor prognosis. In studies such as CLEOPATRA and other real-world studies, over 10% of patients treated with pertuzumab and trastuzumab experienced CNS metastases as the first site of relapse without systemic progression.^5,6^ In our study, 16.9% (20/118) of patients experienced CNS progression during the first-line therapy, and 13.6% (16/118) confirmed CNS as the sole site of progression. This high frequency of CNS progression may be partly related to the lower efficacy of anti-HER2 antibodies such as pertuzumab and trastuzumab in CNS disease.^6^ The median time from the start of first-line therapy to CNS progression was 15.0 months. This finding underscores the need for active CNS surveillance by oncologists, particularly for symptomatic patients. The majority (16/20 patients) with CNS progression switched to pyrotinib-based systemic treatment. Pyrotinib, a novel HER2-specific tyrosine kinase inhibitor (TKI), has shown promising activity for CNS metastasis^7^ and is highly available in China.^8^ Additionally, recent advancements in HER2-targeted therapies have further expanded treatment options for CNS metastases. New TKIs such as neratinib and tucatinib, along with novel HER2-targeted antibody-drug conjugates (ADCs) like Trastuzumab deruxtecan (T-DXd),^9^ have significantly improved the efficacy and provide more treatment options for patients with CNS metastasis.

The toxicity profiles of the combination regimens based on trastuzumab plus pertuzumab were similar to previous clinical trials and real-world data. No new safety signals were identified within this real-world cohort. Although the safety outcomes in our study were consistent with prior findings, it is important to note that the evaluation of toxicities was quite limited by the retrospective nature of this study.

The strengths of our retrospective study are the inclusion of a diverse patient population treated in a real-world clinical setting, which complements findings from restrictively selected populations of clinical trials. To the best of our knowledge, this is the first study to evaluate the equivalence of HLX02 and Herceptin in combination with pertuzumab in the metastatic setting. Additionally, it provides detailed analyses of CNS progression, including incidence rates and treatment patterns.

However, potential limitations should be considered when interpreting the results. First, the OS data are not yet mature due to the relatively short follow-up duration. Second, due to the retrospective nature of the study, the frequency and methods of tumor evaluation were not standardized or different in some patients, when PR/CR was determined, it couldn’t be confirmed if the interval between the next evaluation was greater than 2 months, and AE evaluation also depended on the detail and completeness of medical records, which leads to the results were less reliable than in prospective studies and clinical trials. Besides, the retrospective and observational nature of this study might introduce selection bias between the HLX02 and Herceptin groups. Therefore, comparisons should be interpreted with caution.

In conclusion, the real-world data demonstrate similar efficacy and safety for HLX02 and RTZ, both combined with pertuzumab and various chemotherapy regimens, as the first-line treatment for HER2-positive advanced breast cancer patients in China. In addition, the PFS was consistent with the CLEOPATRA trial for both groups, with no new safety signals, which suggested that HLX02 may provide another option of Her2-targeted therapy combined with pertuzumab for Chinese patients. The benefit regarding long-term survival need to be further verified.

## Data Availability

The data underlying this article will be shared on reasonable request to the corresponding author.
